# Simulations to Assess the Performance of Multifactor Risk Scores for Predicting Myopia Prevalence in Children and Adolescents in China

**DOI:** 10.3389/fgene.2022.861164

**Published:** 2022-04-11

**Authors:** Hong Wang, Liansheng Li, Wencan Wang, Hao Wang, Youyuan Zhuang, Xiaoyan Lu, Guosi Zhang, Siyu Wang, Peng Lin, Chong Chen, Yu Bai, Qi Chen, Hao Chen, Jia Qu, Liangde Xu

**Affiliations:** ^1^ School of Biomedical Engineering, School of Ophthalmology and Optometry and Eye Hospital, Wenzhou Medical University, Wenzhou, China; ^2^ Center of Optometry International Innovation of Wenzhou, Wenzhou, China; ^3^ Wenzhou Realdata Medical Research Co., Ltd, Wenzhou, China; ^4^ Wenzhou PSI Medical Laboratory Co., Ltd, Wenzhou, China

**Keywords:** myopia, multifactor, correct posture, age, assembled myopia predictor

## Abstract

**Background:** Myopia is the most common visual impairment among Chinese children and adolescents. The purpose of this study is to explore key interventions for myopia prevalence, especially for early-onset myopia and high myopia.

**Methods:** Univariate and multivariate analyses were conducted to evaluate potential associations between risk factor exposure and myopia. LASSO was performed to prioritize the risk features, and the selected leading factors were used to establish the assembled simulation model. Finally, two forecasting models were constructed to predict the risk of myopia and high myopia.

**Results:** Children and adolescents with persistently incorrect posture had a high risk of myopia (OR 7.205, 95% CI 5.999–8.652), which was 2.8 times higher than that in students who always maintained correct posture. In the cohort with high myopia, sleep time of less than 7 h per day (OR 9.789, 95% CI 6.865–13.958), incorrect sitting posture (OR 8.975, 95% CI 5.339–15.086), and siblings with spherical equivalent <−6.00 D (OR 8.439, 95% CI 5.420–13.142) were the top three risk factors. The AUCs of integrated simulation models for myopia and high myopia were 0.8716 and 0.8191, respectively.

**Conclusion:** The findings illustrate that keeping incorrect posture is the leading risk factor for myopia onset, while the onset age of myopia is the primary factor affecting high myopia progression. The age between 8 and 12 years is the crucial stage for clinical intervention, especially for children with parental myopia.

## Introduction

The global prevalence of myopia has been rising at an astonishing speed, with nearly 30% of the world population currently having myopia (Dolgin, 2015; Morgan et al., 2018; Sankaridurg et al., 2021). It is anticipated that this number may further rise and even reach one-half of the global population in 2050 (Holden et al., 2016; Sankaridurg et al., 2021). At the same time, almost 10% of the world population is expected to experience high myopia, which will cause much more severe visual impairment and become an unbearably heavy burden for both individuals and society (Williams et al., 2015b; Sankaridurg et al., 2021). According to the latest statistics, the ratio of Chinese children and adolescents suffering from myopia has already reached 52.7%, which makes things even more frightening (Ma et al., 2016; Mountjoy et al., 2018). In particular, owing to the global outbreak of COVID-19 in 2019, children and adolescents were forced to learn at home through screen teaching and were deprived of outdoor activities, which tremendously increased their risk of myopia (Xu et al., 2021a; Xu et al., 2021b). With the advent of the epidemic era, intermittent home quarantine will become the norm. It has become an urgent need to thoroughly investigate the epidemiological characteristics of myopia, comprehensively explore the risk factors of myopia, and formulate feasible and effective prevention strategies.

Numerous epidemiologic studies have been conducted to explore risk factors associated with myopia (Morgan et al., 2021; Sankaridurg et al., 2021). It has frequently been reported that outdoor time (Wu et al., 2013), reading time (Liang et al., 2021), diet habits (Burke et al., 2021), parental myopia, and education level were closely related to myopia (Jan et al., 2019; Jiang et al., 2020; Morgan et al., 2021). A retrospective myopia study between 1983 and 2017 indicated that the main risk factors for increasing prevalence of schoolchildren myopia were older age and more time spent on near-work (Tsai et al., 2021). It has also been verified by multiple studies that education level and outdoor time were two major risk factors for students’ myopia at present (Morgan et al., 2018 ; Mountjoy et al., 2018). Cordain et al. suggested that diet habits could be a possible reason for the increased prevalence of myopia (Morgan et al., 2021). Studies of different ethnic groups have shown that parental myopia, either father’s or mother’s, increased the risk of myopia in their children (Ghorbani Mojarrad et al., 2018; Morgan et al., 2021). However, several reports have only focused on a few risk factors and lacked a sufficiently large sample size. Moreover, high myopia was often neglected. Therefore, it is necessary to recruit sufficient participants and develop novel and effective predicting models for myopia and high myopia by taking into account the influence of comprehensive risk factors.

In this study, we collected and evaluated multiple risk factors for myopia and high myopia in a large-scale cohort with more than 20,000 children and adolescents. Then, two risk forecasting models were established to predict the prevalence of myopia and identify key interventions for improving the universal visual health.

## Materials and Methods

### Participants

The study protocol was approved by a large-scale survey, CAMS, in Wenzhou, China, as described previously (Xu et al., 2021b). Two years (six times) of follow-up were completed from 2019 to 2021. Participants in our study consisted of 24,318 children and adolescents who were sampled from CAMS using a simple randomized sampling method. The participants with dysgnosia or from a special education school, with abnormal basic information (ID card recording errors, manually entered data, and conflicting questionnaire information), hyperopia (strabismus or others), using orthokeratology or having undergone eye surgery, and missing questionnaires were removed. The study protocol and recruitment method were approved by the Ethics Committee of the Eye Hospital Affiliated to Wenzhou Medical University (2021-015-K-21). All procedures were conducted in accordance with the ethical standards of the institution/National Research Council and the 1964 Helsinki Declaration.

### Examination and Groups

All students underwent a comprehensive eye examination, and the detailed procedure was described by CAMS. Distance visual acuity (DVA) was assessed using a Chinese standard logarithm visual acuity E chart (GB 11533-2011) in an illuminated room (WSVC-100, Wenzhou, China). Non-cycloplegic autorefraction was conducted using Goaleye RM-9000 (Shenzhen Aist Industrial Co., Ltd., China). We calculated the spherical equivalent (SE) *via* the sphere plus one half of the cylinder. Emmetropia was defined as +0.25 D <SE ≤−0.25 D in both eyes. Common myopia was defined as −6.00 D <SE ≤−0.50 D, and high myopia was defined as SE ≤−6.00 D. All individuals with common myopia and high myopia were classified under myopia. Students were randomly grouped into the training cohort and the internal validation cohort at a ratio of 7:3. In addition, the prognostic cohort with six follow-up eye biometries was used to identify the survival effects of myopia-related risk factors.

### Questionnaires

Parents completed questionnaires about the demographics, behaviors, and family background of their children. The following information related to myopia was available from the questionnaires. First, demographic data, including age, height, weight, gender, delivery way, school type, school region, vision condition, and onset age of myopia. Second, behaviors, including correct posture, reading time, screen time, outdoor time, sleep time, taste, and diet habits. The correct posture means that students keep their eyes one foot away from the book, a punch from their chest, and one inch from the tip of their pen when writing. The indicator of correct posture was determined by how many of the 10 parental memories of the child’s sitting position met the standard (always, 8–10; basic, 5−7; occasionally, 2−4; never, 0–1). The reading time included time of near-work for learning or pleasure and doing homework. The screen time included time spent on learning or recreational activities on electronic devices, such as computers, televisions, or smartphones. The outdoor time comprised time spent doing activities such as sports, playing, or walking outside. Third, family background, comprising the information regarding parental education and refractive status of family members. Considering that other eye diseases also affect refractive status, “other eye diseases” was listed as a subgroup of refractive status of family members.

### Evaluation of Risk Factors With LASSO Penalty

A total of 18 ocular clinical features and biological parameters related to myopia were extracted from the self-designed questionnaire, which were assigned as risk factors. We employed the least absolute shrinkage and selection operator (LASSO) algorithm to assess and rank the effect size of these risk features of myopia (Gao et al., 2020; Liang et al., 2020). The R package glmnet was utilized to calculate the risk score of each factor with statistical significance (Gao et al., 2020; Liang et al., 2020). The two key parameters of glmnet were set as *family =* “*binomial*”(logistic regression) and *α = 1*(lasso regression). The formula used is as follows: $J\left(\beta_{0},\;\beta \right)=\underset{\;\left(\beta_{0},\;\beta \right)\in {\mathbb{R}}^{p+1}}{\min}-\left[\frac{1}{N}\sum_{i=1}^{N}y_{i}\cdot \left(\beta_{0}+x_{i}^{T}\beta \right)-\text{log}\left(1+e^{\beta_{0}+x_{i}^{T}\beta }\right)\right]+\text{ λ}\left[\left\|\beta_{1}\right\|\right]$ , where *N* is the size of the training cohort; $\left(\beta_{0},\;\beta \right)$ is a parameter vector with length *p*+1; λ is the regularization parameter; $\beta_{1}$ represents the L1 norm of the parameter β ; $x_{i}$ are the feature values for sample *i*; and $y_{i}$ is the real phenotypic category of sample *i*.

In particular, we performed a ten-fold cross-validation on the training cohort to calculate the weight vectors of LASSO penalty (expressed as lambda; the key parameters of cv. glmnet were set as *type. measure =* “*auc*” and *nfolds = 10*). The lambda with 1 SE of the minimum partial likelihood deviance was used for risk factor evaluation and prioritization (Liang et al., 2020).

### Machine Learning for Survival Modeling

We trained the models to predict the risk of myopia (or high myopia) with the nine (or eight) features selected by LASSO. At the beginning, we fitted four candidate machine learning (ML) models, including logistic regression (LR) (Meurer and Tolles, 2017), random forest (RF) (Lin et al., 2018), gradient boosted decision tree (GBDT) (Zou et al., 2019), and neural network (NN) (Tan et al., 2021), into the myopia or high myopia cohort. Ten-fold cross-validation was used to fine-tune the model parameters. The training cohort was standardized by using Box-Cox, Center, and Scale of R package (Tan et al., 2021). During the procedure, weighted cross-entropy (myopia: 0.4 and high myopia: 0.65) was adapted to the unbalanced samples to increase the penalty for misclassified categories and to improve the accuracy and detection rate (Yu et al., 2019).

Subsequently, an ensemble model derived from 3 ML models with best predictive performance (LR, GBDT, and NN) was proposed by weighted voting. Specifically, the risk effects of myopia (high myopia) calculated in LR, GBDT, and NN were considered to assign weights. LR, GBDT, and NN in the integrated model were defined as 0.4 (0.1 for high myopia), 0.5 (0.7), and 0.1 (0.2), respectively. The R package of Caret was used for model training. The validation cohort was adopted and evaluated independently for the performance measuring, including areas under the ROC curve (AUC), accuracy (ACC), sensitivity (SE), specificity (SP), positive predictive value (PPV), and negative predictive value (NPV).

### Statistical Methods

Measurements only from the right eye were used for the analyses. Medians (standard deviation, SD) and frequencies (%) of continuous and categorical variables were assessed, respectively. Continuous variables were compared using the Kruskal−Wallis rank-sum test (tableOne). The odds ratios (ORs) and the corresponding 95% confidence intervals (CIs) were used to evaluate the associations between risk factors and myopia prevalence (STATS). Univariate and multivariate Cox regression analyses were utilized to calculate the hazard ratios (HRs, survival). A nomogram was constructed to plot the factor effects on the onset and development of myopia (rms). Modeling performance was calculated using pROC and Caret. Survival curves were developed using the Kaplan–Meier method with log-rank test and plotted using survival and survminer. Values of *p* < 0.05 were considered statistically significant. Data analyses were conducted using R software (version 4.0.2).

## Results

### Cohort Demographic Data

**FIGURE 1:**
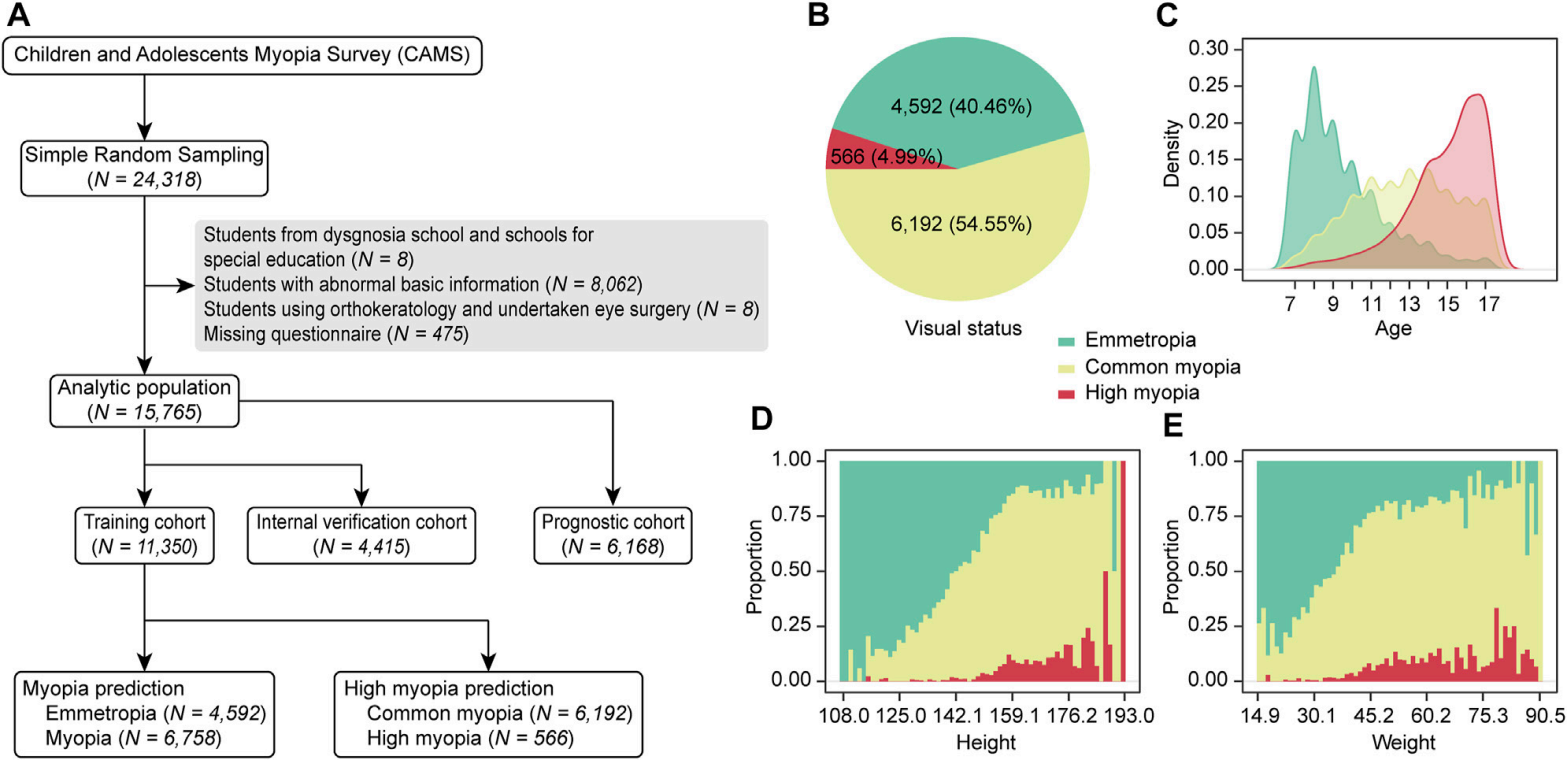
Flowchart and distribution of the study cohort. **(A)** Numbers of participants. **(B)** Distribution of visual states in our cohort. **(C)** Age distribution of schoolchildren with various visual states. **(D)** Height distribution. **(E)** Weight distribution.

Participants in this study included 24,318 children and adolescents, involving 12 regions, 680 primary schools, 281 junior high schools, and 143 senior high schools. After quality control, we excluded from the analyses all those with special education, incorrect biometry, preexisting eye conditions (e.g., orthokeratology and eye surgery), and missing questionnaires (Figure 1A). The remaining 15,765 (65%) students aged 6–18 years were assigned at a ratio of 7:3 to the training cohort (n = 11,350 students) and the internal validation cohort (n = 4,415 students). In addition, 6,168 students with six times of follow-up from 2019 to 2021 were assigned to the prognostic cohort for further survival modeling. In the training cohort, there were 4,592 (40.46%) students with normal vision, 6,192 (54.55%) students with common myopia, and 566 students (4.99%) with high myopia. The characteristics of students with and without myopia are shown in Supplementary Table S1. Overall, the median SE of the myopia group and the high myopia group were −2.88 D (IQR −4.25 D, −1.88 D) and −7.12 D (IQR −8.00 D, −6.50 D), respectively. The median age of students with emmetropia was 9 years (IQR 8–11), while the median age of those with myopia was 13 years (IQR 11–15). The median height of individuals with emmetropia was 135.1 cm (IQR 128.0–147.0 cm), while the median height of those with myopia was 159.0 cm (IQR 147.5–166.0 cm). Children and adolescents with myopia were more likely to be older than 10 years and to be precocious than the children and adolescents with emmetropia. Specifically, the trend increased with the progression of myopia (Figures 1C–E). The proportion of girls with myopia was 51.8%. A total of 1,107 (16.4%) children and adolescents with myopia failed to correct vision in time. In addition, 779 students (11.5%) presented other ocular problems such as strabismus and amblyopia.

### Characteristics of Risk Factors Associated With Myopia

**FIGURE 2:**
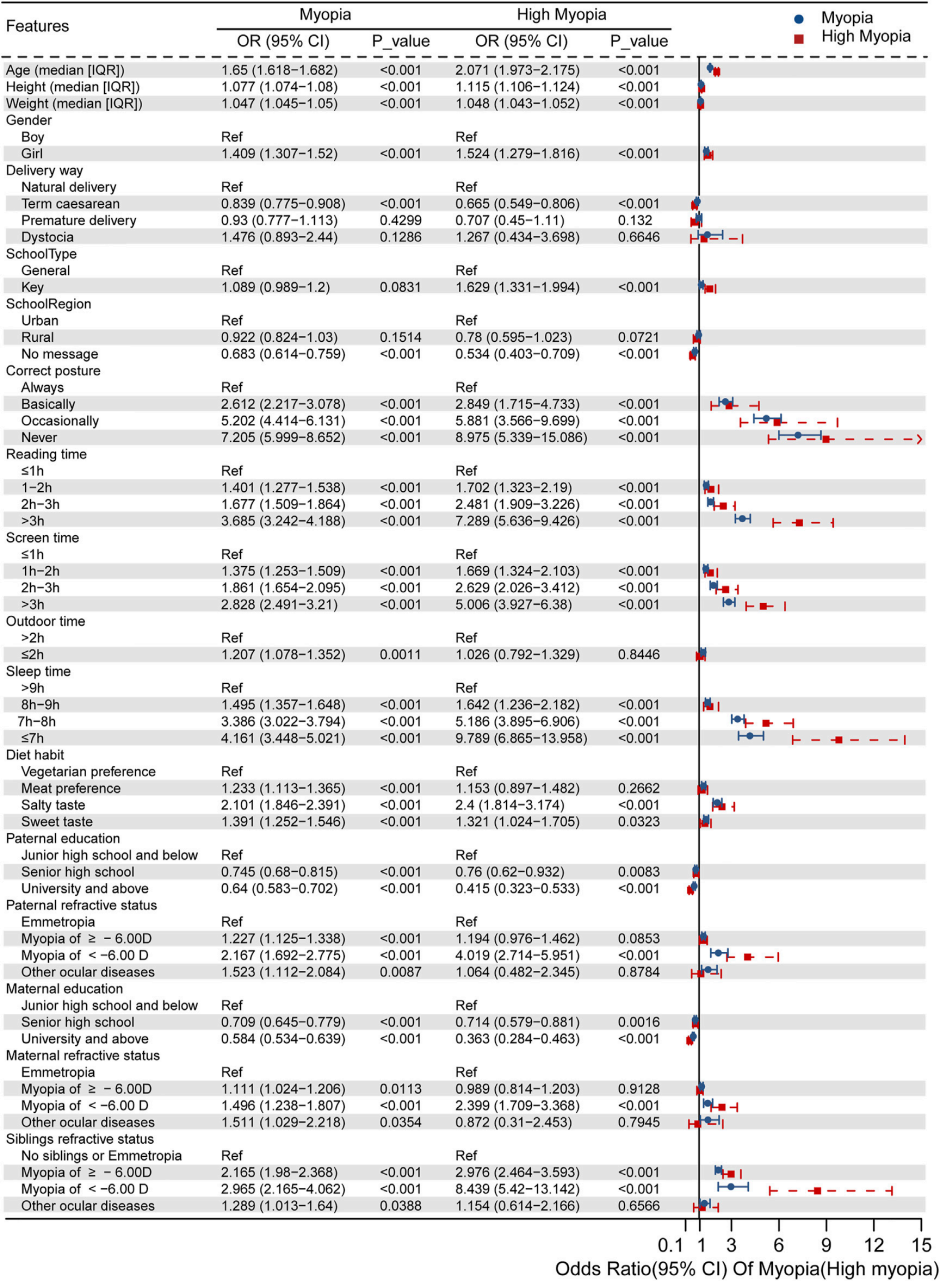
Independent risk factors for myopia or high myopia evaluated *via* univariate regression analysis.

We grouped the risk factors into three categories, consisting of demographic data, behaviors, and family background (Methods). In the group of demographic data, the univariate regression analysis indicated that age (OR 1.650, 95% CI 1.618–1.682, *p* < 0.001) and female sex (OR 1.409, 95% CI 1.307–1.520, *p* < 0.001) were the risk factors for myopia, while full-term caesarean delivery (OR 0.839, 95% CI 0.775–0.908, *p* < 0.001) was a protective factor (Figure 2). Students suffering from high myopia showed similar risk factors in terms of demographic features, and key school (OR 1.629, 95% CI 1.331–1.994, *p* < 0.001) was also a risk factor. However, age exhibited much higher effect size in the high myopia group (OR 2.071, 95% CI 1.973–2.175, *p* < 0.001). Also, there is no difference in vision status between students in rural schools and urban schools (myopia OR 0.922, 95% CI 0.824–1.03, *p* = 0.1514; high myopia OR 0.78, 95% CI 0.595–1.023, *p* = 0.0721).

When focusing on daily behaviors, we found that incorrect posture, more time spent on reading and electronic products, and shorter outdoor and sleep time increased the risk compared with keeping healthy living habits. Importantly, persistently incorrect posture was the leading risk feature for myopia (OR 7.205, 95% CI 5.999–8.652, *p* < 0.001) and high myopia (OR 8.975, 95% CI 5.339–15.086, *p* < 0.001). All of the measured parameters were worse in the high myopia group, except for the outdoor time <2 h per day and meat preference, which were not significant.

Finally, as for family background, we considered both the parental education and heritability of the refractive status (mother, father, and siblings). Higher level of parental education was a protective factor for both myopia (OR for father 0.640, 95% CI 0.583–0.702, *p* < 0.001; OR for mother 0.584, 95% CI 0.534–0.639, *p* < 0.001) and high myopia (OR for father 0.415, 95% CI 0.323–0.533, *p* < 0.001; OR for mother 0.363, 95% CI 0.284–0.463, *p* < 0.001). Interestingly, the refractive status of family members presented dominantly negative effects. Having parents and siblings with shortsightedness or other ocular diseases could affect the child’s myopia. Children whose family members had SE <−6.00 D were more likely to have high myopia (OR for paternal high myopia 4.019, 95% CI 2.714–5.951, *p* < 0.001; OR for maternal high myopia 2.399, 95% CI 1.709–3.368, *p* < 0.001; and OR for siblings’ high myopia 8.439, 95% CI 5.42–13.124, *p* < 0.001).

### Construction of Myopia Risk Prediction Model

We conducted a multifactor evaluation using SE of students with myopia or high myopia in the training cohort and related environmental factors for feature selection. Among 20 risk features extracted from the questionnaires, we selected nine features by LASSO to construct the myopia risk prediction model (Supplementary Figures S1A,B). The effect size of correct posture, age, paternal refractive status, maternal refractive status, siblings’ refractive status, gender, reading time, outdoor time, and height was quantified (Supplementary Figure S1E). These factors positively correlated with the onset and development of myopia. Furthermore, 11 features were eventually chosen for high myopia modeling (Supplementary Figures S1C,D), of which seven features, including onset age of myopia, age, siblings’ refractive status, paternal refractive status, maternal refractive status, reading time, and correct posture, were the risk factors, whereas the delivery way, school type, paternal education, and maternal education were the protective features, exhibiting a negative association with high myopia (Supplementary Figure S1F).

**FIGURE 3:**
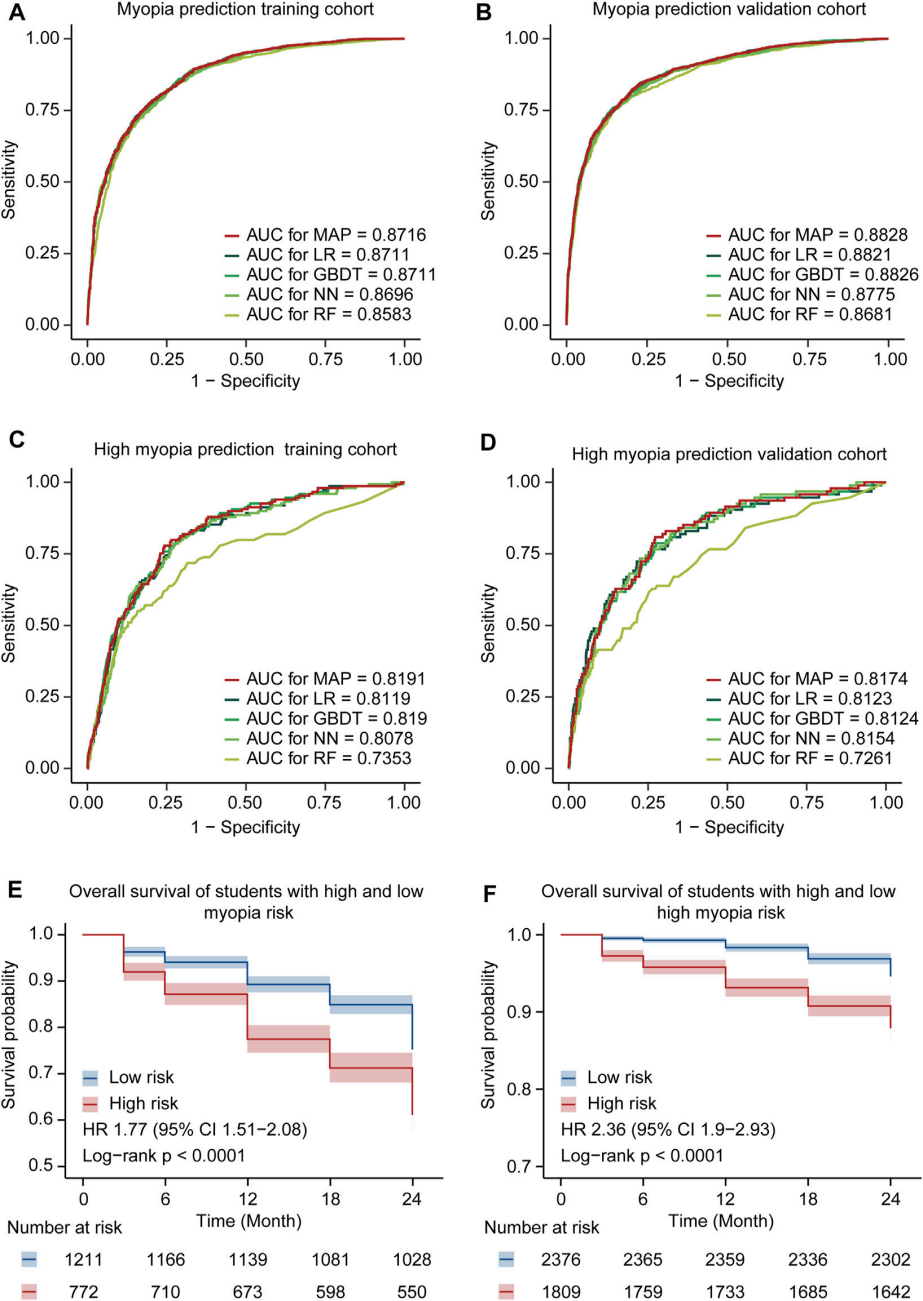
Model performance comparison. **(A)** Comparison of ROC curves for the MAP model and the other ML models of myopia prediction in the training cohort. **(B)** Comparison of ROC curves for the MAP model and the other ML models of myopia prediction in the validation cohort. **(C)** Comparison of ROC curves for the MAP model and the other ML models of high myopia prediction in the training cohort. **(D)** Comparison of ROC curves for the MAP model and the other ML models of high myopia prediction in the validation cohort. **(E)** The Kaplan–Meier curves for developing myopia among students in different risk groups. Shaded areas indicate 95% confidence intervals. **(F)** The Kaplan–Meier curves for developing high myopia among students in different risk groups.

In general, 4 ML models, including LR, GBDT, NN, and RF, were created to evaluate the myopia prevalence and risk of further deterioration. ML algorithms represented slight diverse performance, but they exhibited potential capability for myopia or high myopia risk prediction (Figure 3). Aiming to establish a more reasonable predictive model with enhanced prognostic significance, we integrated the top three methods (LR, GBDT, and NN) to build an ensemble model, that is, myopia assessment and predictor (MAP). As expected, MAP showed better performance in predicting risks of myopia and high myopia than the other four baseline models.

MAP achieved an AUC of 0.8716 (95% CI 0.8598–0.8835) with an accuracy of 79.74% (95% CI 78.35–81.07%) for the identification of myopia in the training cohort. In the validation cohort, MAP demonstrated an AUC of 0.8828 (95% CI 0.8724–0.8931) and an accuracy of 82.06% (95% CI 80.9–83.18%) to predict the onset of myopia. Meanwhile, the AUC and accuracy of MAP were 0.8191 (95% CI 0.7848–0.8533) and 64.33% (95% CI 62.2–66.42%) in the training cohort of high myopia and 0.8174 (95% CI 0.7725–0.8624) and 77.21% (95% CI 75.09–79.22%) in the validation cohort of high myopia, respectively. In addition, the other valuable indexes (SE, SP, PPV, and NPV) of the models holding similar higher performance are listed in Supplementary Table S2 .

Moreover, we extracted the prognostic cohort according to the follow-up survey from June 2019 to June 2021 in CAMS. These six time-point eye biometries were utilized for Kaplan–Meier (survival) analysis, aiming to further confirm the availability and robustness of the ensemble MAP. We calculated the risk of each student in the entire prognostic cohort and divided all patients into low- and high-risk groups. The Kaplan–Meier curves of MAP demonstrated that the low- and high-risk groups were remarkably separated (Figures 3E,F), with a HR of 1.77 (95% CI 1.51–2.08) and 2.36 (95% CI 1.9–2.93), respectively. These findings highlighted the ability of the integrated model to accurately predict and assess the prevalence of myopia and high myopia.

### Risk of Myopia Is Associated With Diverse Environmental Exposure

**FIGURE 4:**
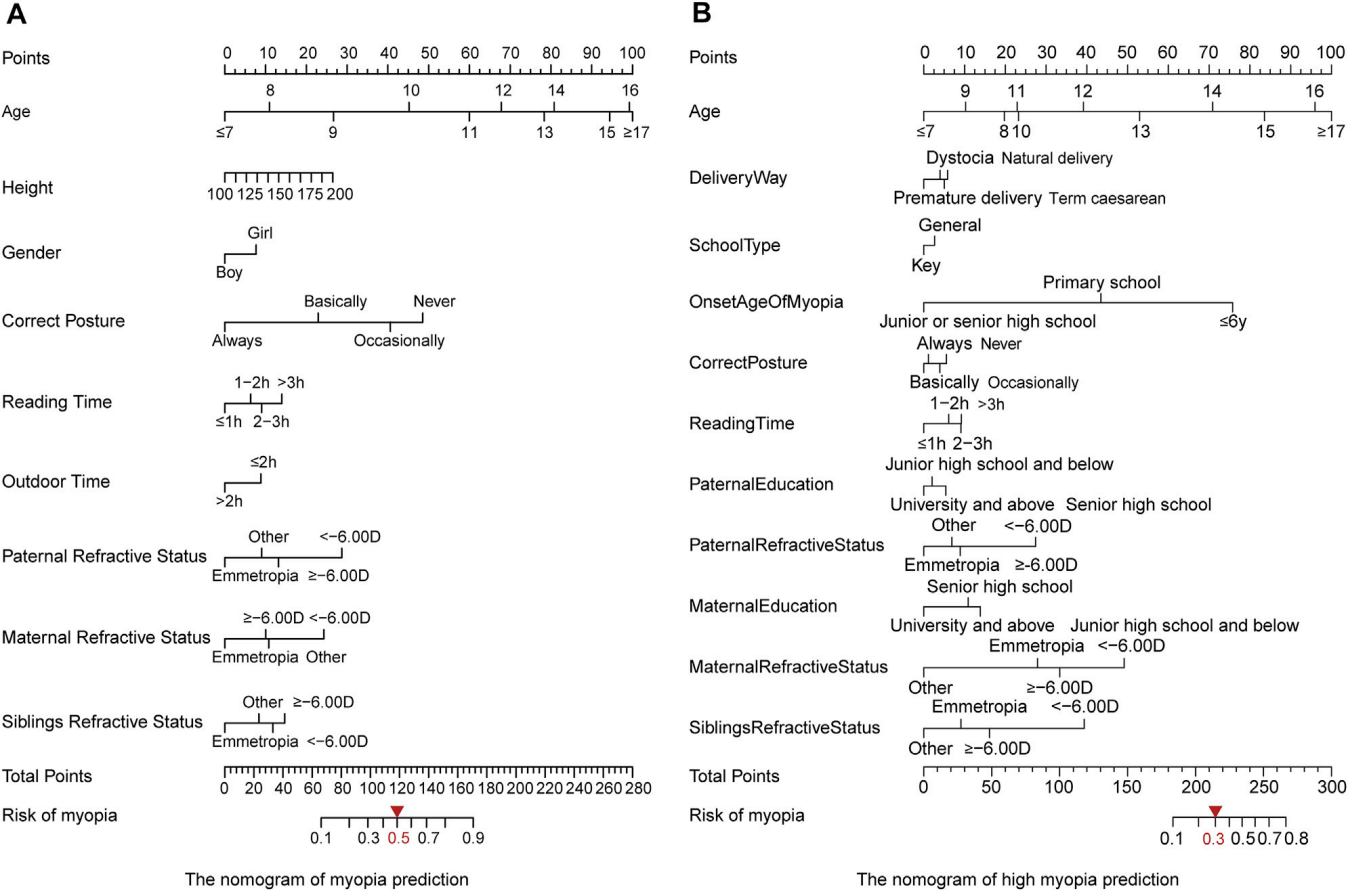
Nomogram of the MAP model to evaluate the prevalence of myopia. **(A)** Myopia modeling. By optimizing the indicators using the training cohort, we set the threshold of 0.5 for assigning the high risk. **(B)** High myopia modeling. The threshold of 0.3 was selected for assigning the high risk.

We further applied the established MAP model to obtain the global insight into myopia onset *via* nomogram. The nomogram is a pictorial representation for depicting the association between risk factors and the probabilities of the focusing events, which provides an intuitive way to interpret the myopia and high myopia prediction model (Figure 4). Each risk factor has a corresponding score on the first row of the nomogram (Points). The greater the score of the factor, the greater its influence on the occurrence of myopia (high myopia). Then, the total score for all risk factors can be calculated. Finally, the risk probability of myopia (high myopia) is obtained through the correspondence between the last two lines of the nomogram (Total Points and Risk of myopia/high myopia). In addition to age, the correct posture and the onset time of myopia were the leading risk factors contributing to the development of myopia or high myopia. Of note, genetics-related aspects, including paternal refractive status, maternal refractive status, and siblings’ refractive status, had more harmful effects on high myopia than on myopia. These findings illustrated that there were different mechanisms in the onset and development of high myopia, where genetic factors might contribute more than environmental features.

## Discussion

In this study, for the first time, we systematically evaluated the risk factors associated with myopia by incorporating ocular biometry and epidemiological factors from more than 20,000 children and adolescents. Based on risk factor selection, we ranked the effect size of the environmental and genetic features influencing ocular status of the included students. Eventually, the ensemble modeling, MAP, was established to prioritize risk factors and predict the probability of myopia occurrence. All these findings suggested that early intervention in living habits, particularly focusing on children’s sitting position, could provide greater benefits for myopia prevention and control, thereby reducing vision loss in myopic children and adolescents.

### Association Between Key Risk Factor Exposure and Myopia

We found that age was an important feature affecting the occurrence of myopia, especially high myopia. The risk of high myopia was negatively associated with the onset time of myopia. Earlier onset time of myopia (≤6 years) was associated with the higher risk of developing high myopia (Supplementary Figure S2). Myopia occurred in primary or junior high schools, resulting in a significant decrease in the proportion of high myopia. Our findings further revealed the current situation of myopia in children and adolescents in China. The early onset of myopia is in coexistence with the growing vision impairment and prevalence of high myopia.

We further investigated the internal mechanism and effect of age on the occurrence and progression of myopia. Previous studies have indicated that the risk of myopia might increase with the development of a child. When the height is stable, the influence of the development on the prevalence of myopia is significantly reduced (Northstone et al., 2013; Azuara-Blanco et al., 2020). However, the detailed relationship and dynamic curve have not yet been depicted. Our study illustrated the height distribution of students with or without myopia at different ages, which helped to identify the key points of myopia prevention and control in the growth period. Supplementary Figure S3 shows the diverse development curves of boys and girls according to our large-scale survey. In girls in the age range from 8 to 12 years, the average height of the myopic students was significantly higher than that of the emmetropia group, representing the right-skewed distribution. Owing to the later development of the boys, skewed distribution of the height of the myopia group was somewhat late; however, at the age of 10–12 years, the average height of the two groups revealed significant difference, that is, the mean height of the myopia group was significantly higher than that of the emmetropia group. We believe that the faster development of children and adolescents is associated with a higher risk of myopia. The age range from 8 to 12 years, in the early stage of development, is the key period for the prevention and control of myopia. It is necessary to pay close attention to the children’s eye health status and check their eyesight regularly.

**FIGURE 5:**
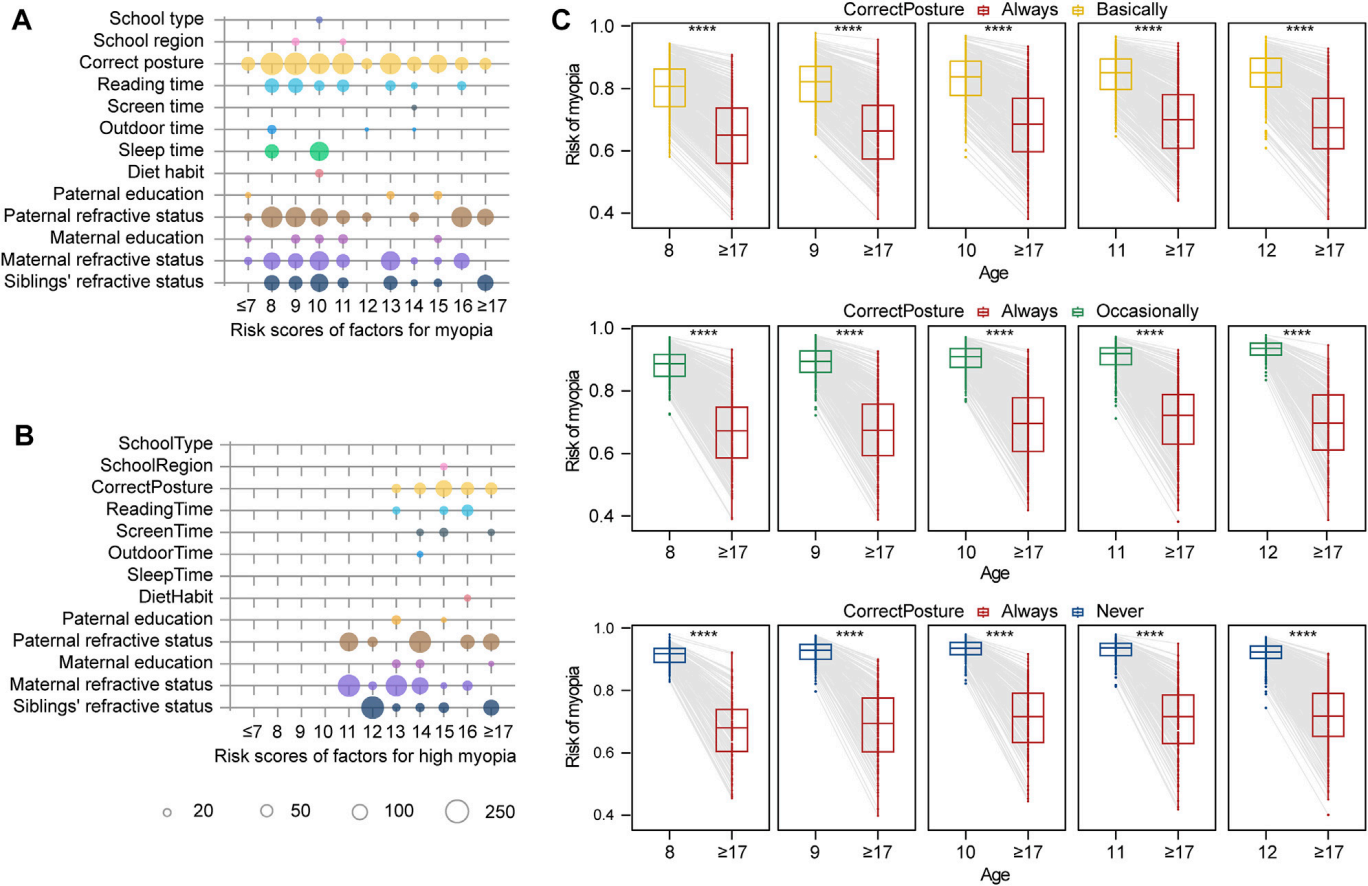
Effect size of factors for myopia risk evaluation and simulated intervention models. **(A)** Risk scores for myopia according to age. The size of the circle indicates the effect size in modeling. **(B)** Risk scores for high myopia according to age. **(C)** Simulation of myopia risk by posture correction for different ages. In the first rectangle, the box chart on the left represents the risk of myopia in 8-year-old children who keep correct posture basically. The box chart on the right represents the risk of myopia in this group of children if they correct their posture at the age of eight and maintain correct posture until the age of 18 years. Four stars representing the difference of myopia risk between two groups are significant, with *p* < 0.0001.

In addition, based on the ranked risk factors by LASSO and the calculated effect scores by the nomogram, correct posture was identified as the leading risk factor in the myopia group, while the onset time of myopia was the key feature in the high myopia group (Supplementary Figure S1). The effects of lifestyle factors and genetic background on myopia also change with age. The effect scores of myopia risk factors at different ages could be obtained, which represented the relationship between risk factor exposure and myopia over time. By drawing the nomogram of the multifactor for each age, we found that the correct posture and family refractive status at the age of 8–12 years exhibited dominant effects on the development of myopia (Figure 5A). We proposed an early intervention model to simulate the risk of myopia by sitting in the correct posture at the age of 8–12 years for the students usually maintaining incorrect posture (Figure 5C). Regardless of the degree of nonstandard sitting posture, the earlier the correct posture was achieved, the more significant decrease in the risk of myopia was observed (p < 0.05, Supplementary Table S3). More importantly, these reported outcomes are the first to highlight the importance of early intervention for correct posture in the prevention and control of myopia in children and adolescents. In particular, students with a family history of myopia should correct their sitting posture at an earlier time and a more critical time, especially at the beginning of primary school (8 years). As for high myopia, the effect of parental myopia was significantly increased, compared with other risk factors (Figure 5B). The dot plot showed that the influence of genetics-related features had a greater impact on high myopia, especially for older age groups (12–14 years).

Previous studies have demonstrated that the level of parental education indirectly affects the probability of myopia in their children (Jiang et al., 2020; Morgan et al., 2021). Namely, higher educational level of parents is associated with a more serious visual loss in children. In contrast, in our study, parental educational level was a protective factor. The proportion of myopia in children and adolescents decreased with the parental education level. Further literature research showed that parents’ attitudes toward the development and control of myopia could have a significant impact on children’s daily habits (McCrann et al., 2018). Myopic parents were more inclined to restrict screen time than nonmyopic parents. Highly educated parents are more likely to cultivate their children’s healthy living habits, thereby generating more positive effects on their children’s visual health. We suspect that previous studies may have confused parental education level with parental myopia, which is a compensating factor. This may be a very prominent compensation effect of environmental factors and genetic risk, which must be taken into account separately.

Furthermore, we found that children delivered by full-term caesarean section were somewhat protected from the onset of myopia (OR 0.839, 95% CI 0.775–0.908, *p* < 0.001), with a lower proportion of myopia occurrence than children born *via* normal delivery (34.30 vs. 60.30%). There was no significant difference among other birth ways. These findings contradict the general perception that “natural birth is the best way for the birth of a child” and are not in agreement with the results of previous studies (Semeraro et al., 2019). However, the emphasis of previous studies was on the influence on myopia in terms of children’s physical condition at birth (weight and whether they were affected by genetic eye diseases or not) (Semeraro et al., 2019; Plotnikov et al., 2020) and their birth time (season, illumination, and social education level) (Williams et al., 2015a) or the impacts of different delivery modes on the eyes of pregnant women with ocular diseases (including myopia) (Chen et al., 2018; Wu et al., 2020). A small number of studies focusing on the effect of birth ways on myopia prevalence in children and adolescents have paid more attention to the factors of preterm birth (Chou et al., 2021). Therefore, it is interesting to study the impacts of diverse birth ways, such as full-term natural birth, full-term caesarean section, and dystocia, on myopia incidence in children and adolescents.

### Strengths and Limitations

One of the advantages of our study is the large sample size, with more than 20,000 participants recruited from CMAS. At the same time, we comprehensively analyzed environmental and genetic factors related to the risk of myopia or high myopia at multiple levels. Compared with previous studies, the integrated ML models were created to predict and simulate the influence of the prioritized risk factors on the onset of myopia. However, some limitations remain to be solved in the future. First, a suitable external validation cohort with sufficient sample size is urgently needed to evaluate the generalization performance of our model. Second, the number of 18-year-old students was insufficient because of the graduation from high school. Finally, some indexes in the questionnaire need to be further optimized.

## Data Availability

The datasets presented in this study can be found in online repositories. The names of the repository/repositories and accession number(s) can be found in the article/Supplementary Material.

## Supplementary Material

Supplementary Figure S1 Feature selection by LASSO. **(A)** The cross-validation curve (red dotted line) along with upper and lower standard deviation curves along the λ sequence (error bars) in the myopia prediction model. Two special values along the λ sequence are indicated by the vertical dotted lines. The left line is the value of λ that gives minimum mean cross-validated error, while the right line is the value of λ that gives the most regularized model such that the cross-validated error is within one standard error of the minimum. **(B)** LASSO variable trace profiles of features whose intracohort missing rates were less than 5% in the myopia prediction model. **(C)** The cross-validation curve along with upper and lower standard deviation curves along the λ sequence in the high myopia prediction model. **(D)** LASSO variable trace profiles of features whose intracohort missing rates were less than 5% in the high myopia prediction model. **(E)** Feature coefficient of LASSO with the best lambda value in the myopia prediction model. Positive coefficient and negative coefficient features are colored in red and blue, respectively. Gray features with coefficient 0 were considered redundant and removed. **(F)** Feature coefficient of LASSO with the best lambda value in the high myopia prediction model.

Supplementary Figure S2 Distribution of high myopia in children and adolescents with different myopia occurrence times. **(A)** Myopia onset at age of ≤6 years. **(B)** Myopia onset at age of primary school. **(C)** Myopia onset at age of junior or senior high school.

Supplementary Figure S3 Height distribution of myopia population at different ages. **(A)** Height distribution of boys aged from 6 to 18 years. Green represents emmetropia, and red represents myopia. The vertical line represents the mean height, and the curve represents the probability density of the height distribution. **(B)** Height distribution of girls aged from 6 to 18 years.
