# Supplementary figures and images for: Simulations to Assess the Performance of Multifactor Risk Scores for Predicting Myopia Prevalence in Children and Adolescents in China
Source: Front Genet. 2022 Apr 11;13:861164. doi: 10.3389/fgene.2022.861164 (PMC9035486; doi:10.3389/fgene.2022.861164)

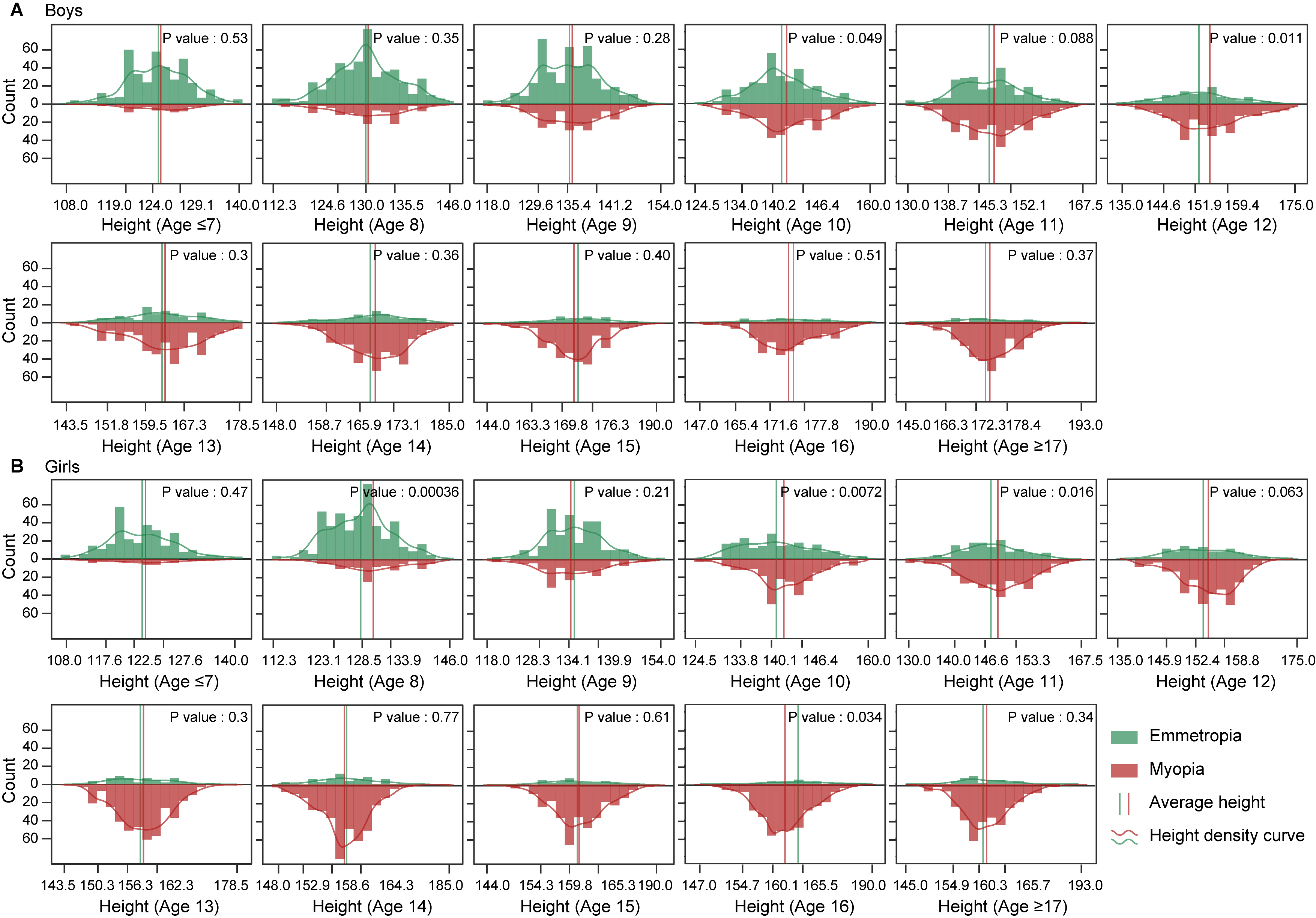

Supplement: Supplementary file 3 [file Image3.TIF]

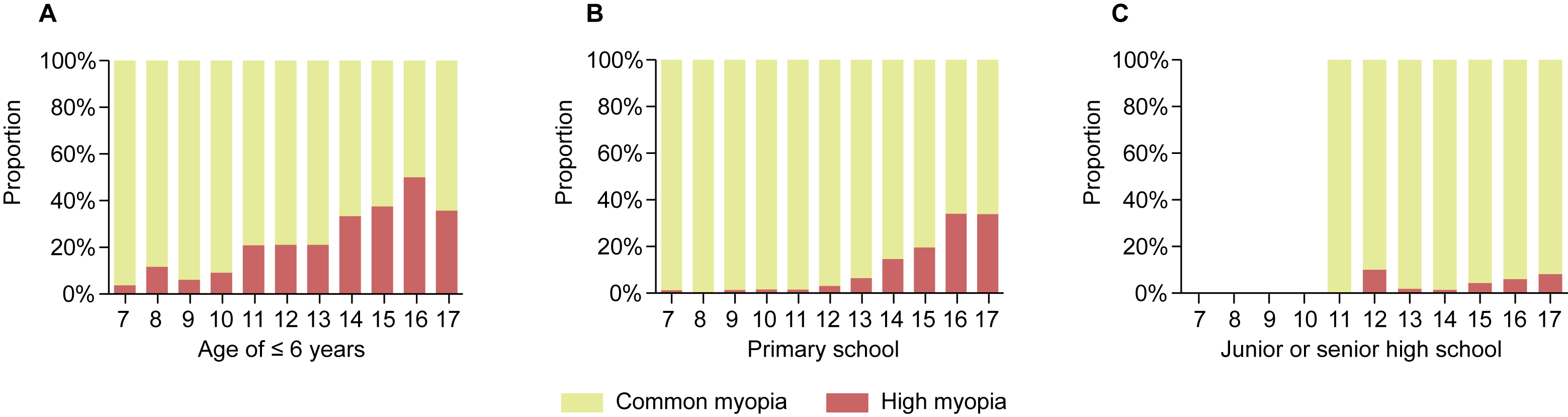

Supplement: Supplementary file 4 [file Image2.TIF]

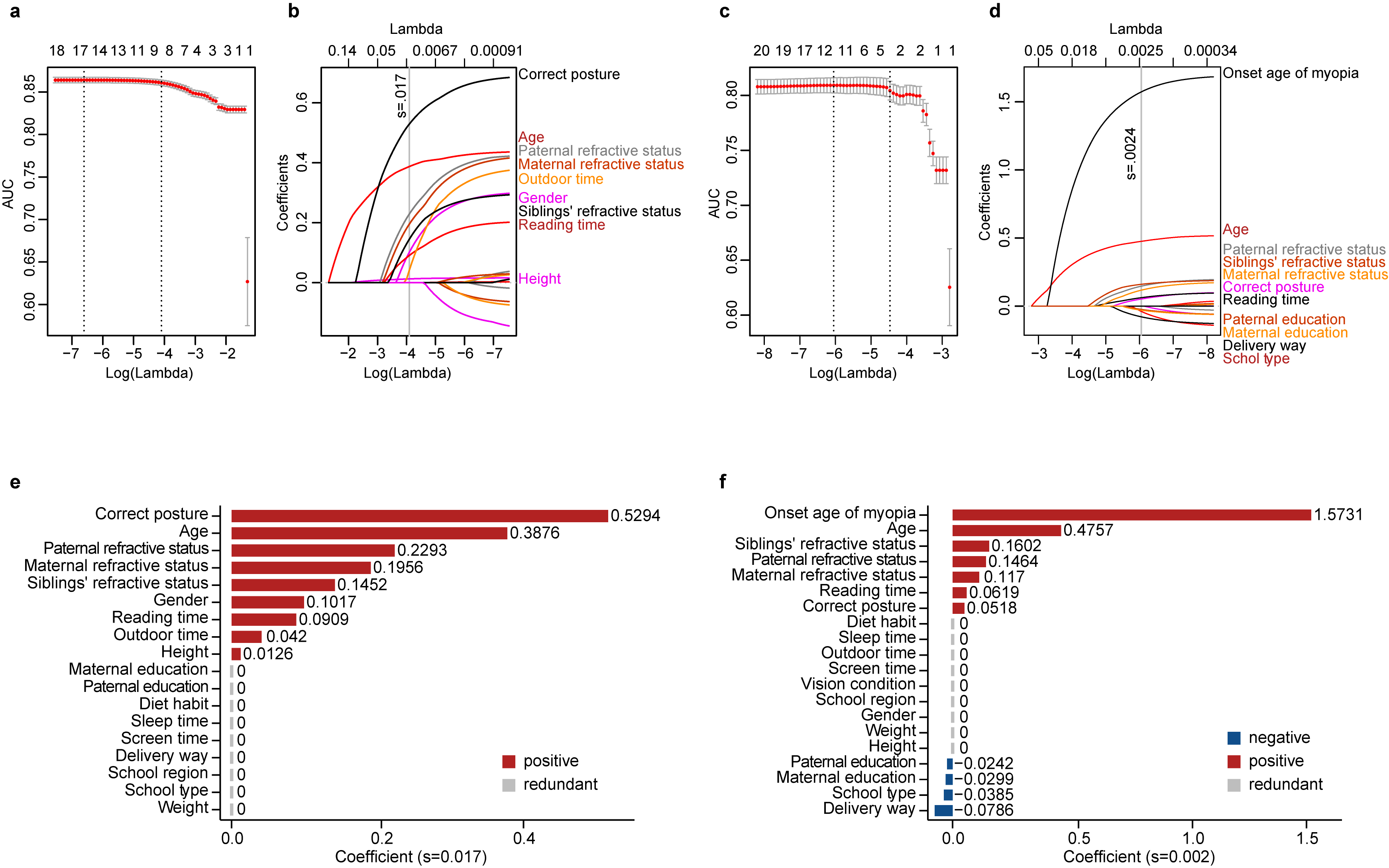

Supplement: Supplementary file 5 [file Image1.TIF]
